# Synergic bactericidal effects of reduced graphene oxide and silver nanoparticles against Gram-positive and Gram-negative bacteria

**DOI:** 10.1038/s41598-017-01669-5

**Published:** 2017-05-08

**Authors:** Karthika Prasad, G. S. Lekshmi, Kola Ostrikov, Vanessa Lussini, James Blinco, Mandhakini Mohandas, Krasimir Vasilev, Steven Bottle, Kateryna Bazaka, Kostya Ostrikov

**Affiliations:** 1 School of Chemistry, Physics and Mechanical Engineering, Queensland University of Technology, Brisbane, Queensland 4000 Australia; 2 CSIRO-QUT Joint Sustainable Materials and Devices Laboratory, Commonwealth Scientific and Industrial Research Organisation, P.O. Box 218, Lindfield, NSW 2070 Australia; 3 Department of Nano science and Technology, Anna university, Chennai, 600 025 India; 4 Institute of Health and Biomedical Innovation, Queensland University of Technology, Brisbane, Queensland 4000 Australia; 5 School of Engineering, University of Southern Australia, Adelaide, South Australia 5000 Australia

## Abstract

Reduced graphene oxide (rGO) is a promising antibacterial material, the efficacy of which can be further enhanced by the addition of silver nanoparticles (nAg). In this study, the mechanisms of antibacterial activity of rGO–nAg nanocomposite against several important human pathogenic multi-drug resistant bacteria, namely Gram-positive coccal *Staphylococcus aureus* and Gram-negative rod-shaped *Escherichia coli* and *Proteus mirabilis* are investigated. At the same concentration (100 µg/ml), rGO–nAg nanocomposite was significantly more effective against all three pathogens than either rGO or nAg. The nanocomposite was equally active against *P. mirabilis* and *S. aureus* as systemic antibiotic nitrofurantoin, and significantly more effective against *E. coli*. Importantly, the inhibition was much faster in the case of rGO–nAg nanocomposite compared to nitrofurantoin, attributed to the synergistic effects of rGO–nAg mediated contact killing and oxidative stress. This study may provide new insights for the better understanding of antibacterial actions of rGO–nAg nanocomposite and for the better designing of graphene-based antibiotics or other biomedical applications.

## Introduction

According to the report published by World Health Organization, bacterial resistance to antibiotics is a major global threat to public health akin that posed by global warming and terrorism^1^. In the European Union alone, annual health care costs and productivity losses attributed to bacterial resistance by major health care-associated bacterial infections is estimated to approach 2.5 million hospital days, 25,000 deaths and economic losses on the order of €1.5 billion^2^. Unsurprisingly, there is a significant interest in the development of novel strategies to combat the spread of resistant microorganisms, e.g. by developing new antibiotics and other therapeutics^3–5^. Alternative therapies that positively contribute to the rational use of conventional antibiotics are particularly highly desired^6^.

Recently, graphene-based materials have emerged as promising antibacterial materials^7–11^. Originally actively researched for their excellent thermal, mechanical and electrical properties that make them well-suited for such applications as energy devices, sensors, and field-effect transistors^12, 13^, chemically modified graphenes such as graphene oxide (GO) and reduced GO (rGO) have been shown to inhibit the growth of several clinically-relevant pathogens, including *Escherichia coli* ^14–16^. The observed antibacterial activity of GO and rGO has been attributed to the favorable combination of physical structure and chemical functionality^17^, where the basal planes and edges of GO are decorated with exogenous functional groups such as hydroxyl, epoxy group and carbonyl groups^18, 19^. Upon contact with such a nanostructure, membrane stress induced by the sharp edges of graphene nanosheets has been shown to cause significant physical damage to cell membrane, and subsequent loss of bacterial membrane integrity and leakage of intracellular material^20^. As the case with other nanomaterials, smaller sized nanoparticles (<10 nm) of rGO were found to exhibit higher antibacterial activity, owing to the favorable combination of high surface area and mobility across cell membrane^21, 22^.

Stronger antibacterial activity can potentially be achieved by combining nanomaterials with complimentary action against multiple bacterial targets^23^. The present study explores whether it is possible to complement membranolytic and oxidative activity of rGO with the free radical formation of silver nanoparticles. The antibacterial properties of silver and silver-based nanomaterials are well-documented^24–26^. The benefits of Ag ions and Ag nanoparticles include their efficacy against both Gram-positive and Gram-negative bacteria, and a multifaceted mechanism of action. This multifaceted mechanism of action translates into attacking the bacteria on several fronts (e.g. blocking respiration by binding to bacterial DNA, binding to enzyme to block energy cycle, binding to protein disulfide bridges to disrupt function), which makes it difficult for bacteria to develop resistance. This gives silver advantages compared to traditional antibiotics which typically target only a single site of the bacterium cell. Importantly, Ag nanoparticles (nAg) show low or no cytotoxicity to human cells and are suggested in some reports that silver nanoparticles aids in reducing inflammation^27–30^. This combination of properties makes silver and silver nanoparticles very attractive in protecting medical devices prone to being infected.

This investigation aims to explore the mechanisms of activity of rGO–nAg nanocomposites against pathogenic multi-drug resistant bacterial species, namely *Escherichia coli* (gram negative), *Staphylococcus aureus* (gram positive), and *Proteus mirabilis* (gram negative).

## Results

### Structural and morphological characterization of nanomaterials

Successful formation of rGO was confirmed by SEM and TEM microscopy.

**Figure 1:**
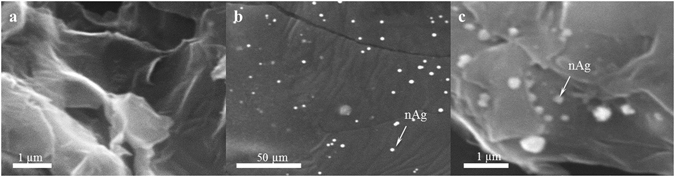
SEM images of synthesized nanomaterials. (**a**) rGO nanosheets with a large number of reactive edges, (**b**) nAg nanoparticles of uniform size and near spherical shape, (**c**) rGO–nAg composite showing uniform distribution of nAg.

The microstructural analysis (Fig. 1a) shows a sheet-like structure with wrinkles, and a relatively large number of reactive edges indicative of the formation of rGO nanoflakes. SEM imaging confirmed a well dispersed solution of silver nanomaterials having approximately spherical shape (Fig. 1b). The rGO dispersion remained homogeneous for several days, which facilitated uniform dispersion and binding of Ag nanoparticles to rGO sheets (Fig. 1c).

**Figure 2:**
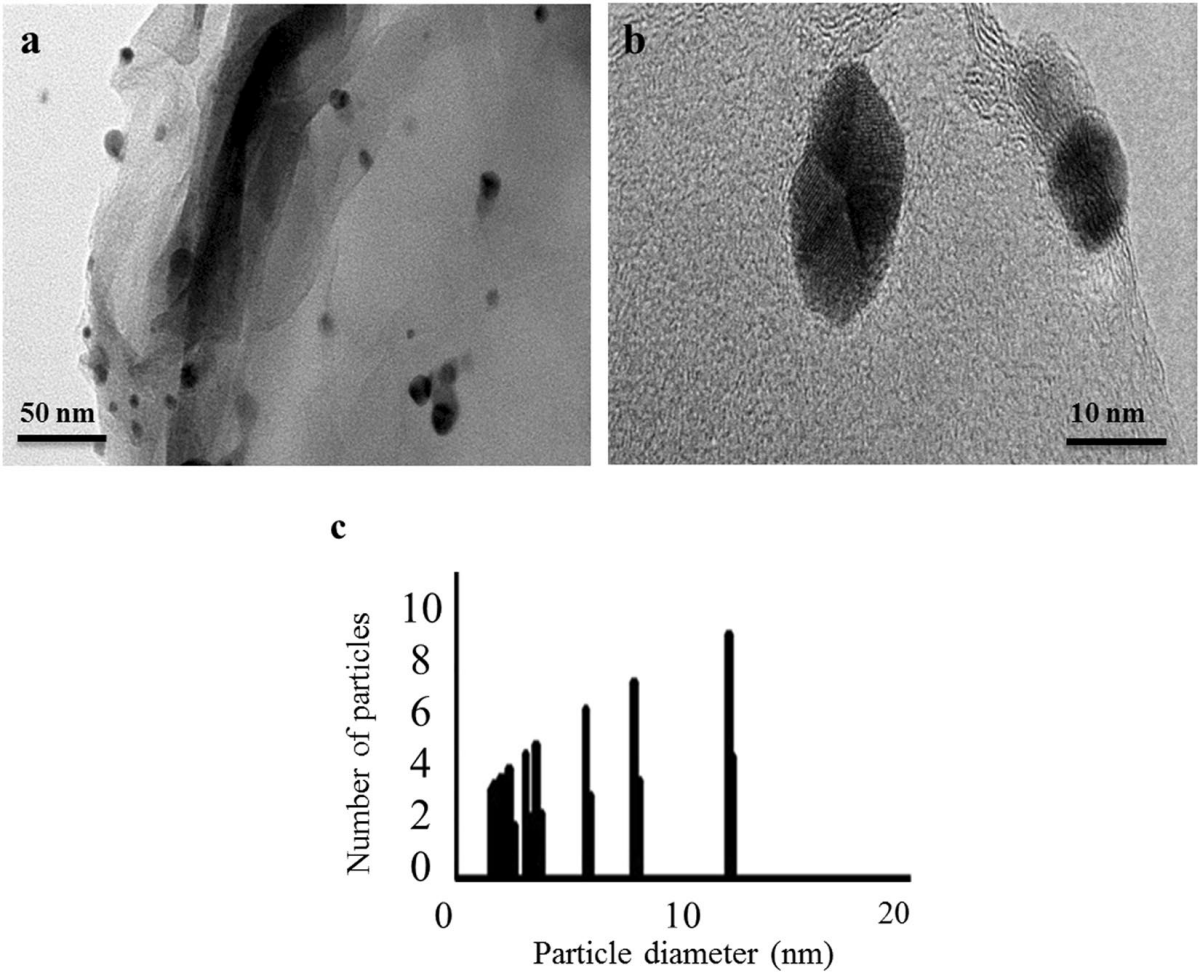
Representative HRTEM images of (**a**) rGO–nAg nanocomposite, (**b**) lattice resolved image of nAg in rGO–nAg nanocomposite. (**c**) Size distribution histogram of nAg in rGO–nAg nanocomposite presented in (**a**).

The morphology of rGO–nAg nanocomposites was examined using high-resolution transmission electron microscopy (HRTEM). The HRTEM images presented in Fig. 2a showed nAg with an average diameter of 5.36 nm to be uniformly distributed on the rGO. The lattice fringes of nAg shown in Fig. 2b confirm the crystalline structure of nAg. The particle size distribution of rGO–nAg nanocomposite (Fig. 2c) was estimated using ImageJ software and HRTEM image shown in Fig. 2a. Based on the size distribution histogram and HRTEM images the size of nAg was in the range of 1–15 nm.

**Figure 3:**
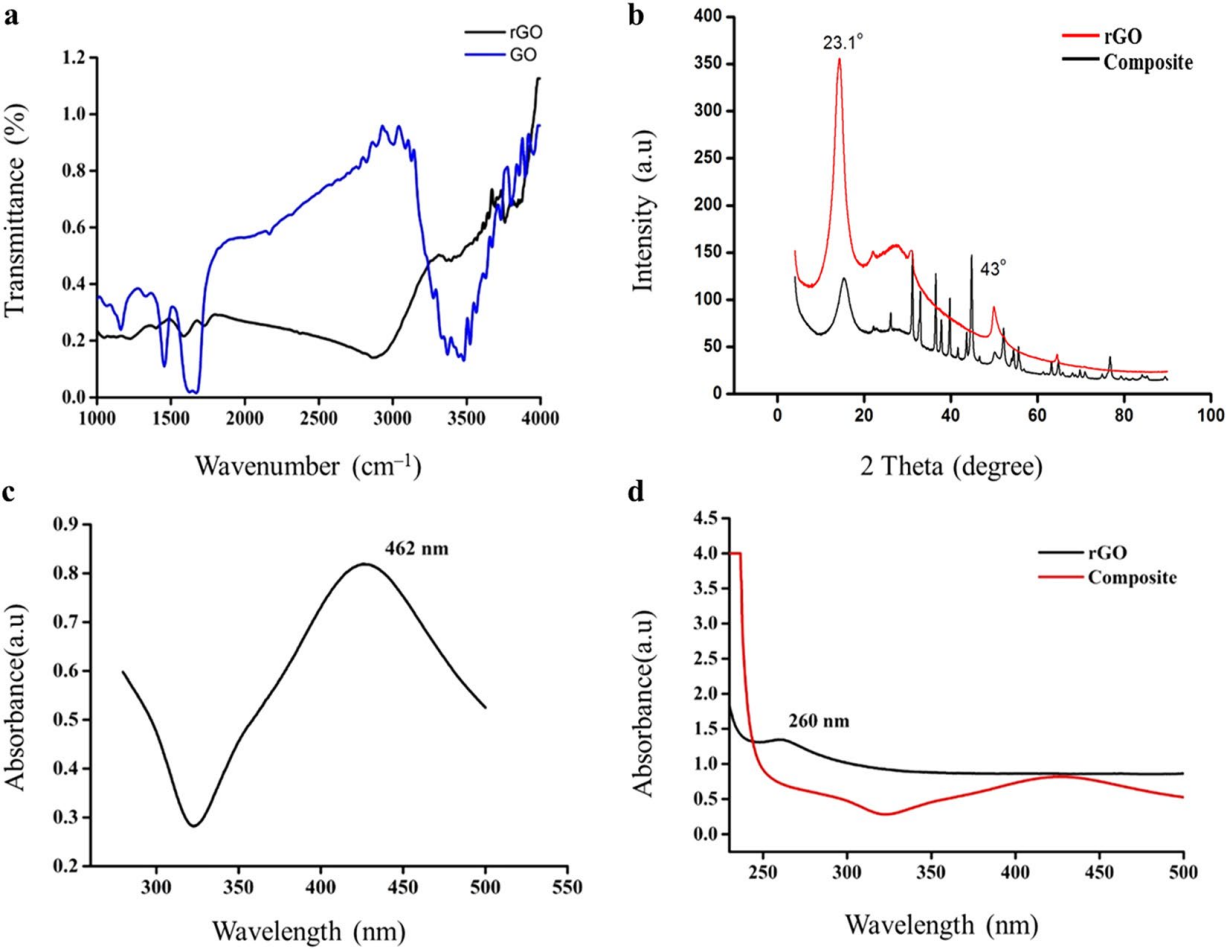
Reduction of GO to rGO and subsequent incorporation of nAg was confirmed spectroscopically: (**a**) FTIR spectra for GO and rGO; (**b**) XRD of rGO and rGO–nAg composite; (**c**) UV spectrum of nAg; (**d**) UV–Vis spectra for rGO and rGO–nAg composite.

The FTIR spectra (Fig. 3a) of rGO significantly differed from that of GO. The peak at 3500 cm^−1^ is typically attributed to O–H stretching vibrations of adsorbed water molecules and structural OH groups, and the peak at 1600 cm^−1^ is attributed to O–H bending vibrations^31, 32^. The presence of carboxyl and epoxy functional groups can also be detected at around 1734 cm^−1^, 1225 cm^−1^ and 1053 cm^−1^, respectively^32^. Due to thermal reduction, some oxygen-containing functional groups are partially removed. The intense absorption band at 3500 cm^−1^ is decreased after reduction. The carboxyl stretching vibration is also decreased. The absorption intensity of the band at 1080 cm^−1^, which is assigned to epoxide (C–O–C) group, is also weakened in reduced graphene oxide^33^.

The investigated structure diffracts the monochromatic beam of x-rays. As can be seen on the spectrum for rGO (Fig. 3b), a new high index, strong broad peak is obtained at 2θ = 24.1° for (002) plane and a small peak is obtained at 2θ = 42.91° for (100) plane. It is the transitional stage between graphene oxide and graphene, as rGO is obtained with a peak value at 2θ = 23.1° for (002) plane and a small index peak of graphene existence is observed at 2θ = 43° for (100) plane with inter layer distance of 0.37 nm^34^. For the rGO–nAg composite, along with the observed diffraction peaks at 2θ = 23, 43°, the XRD pattern also showed peaks at 38°, 46° and 64°, which according to the JCPDS files 04-0783 and 84-0713, correspond to (111), (200) and (220) crystal planes of nAg. This confirms the formation of rGO–nAg composite.

The addition of nAg particles to rGO produces a characteristic absorption band at 426 nm (Fig. 3c,d). That is, an intense longitudinal band has appeared due to the contribution from the dipole oscillation along the long axis of the nanomaterials^35^. The rGO–nAg formation was visually confirmed as a continuous color change of the solution from light yellow to gray. The UV-Vis spectroscopy of rGO showed a peak red-shifted to 260 nm, confirming successful GO reduction (Fig. 3d).

**Figure 4:**
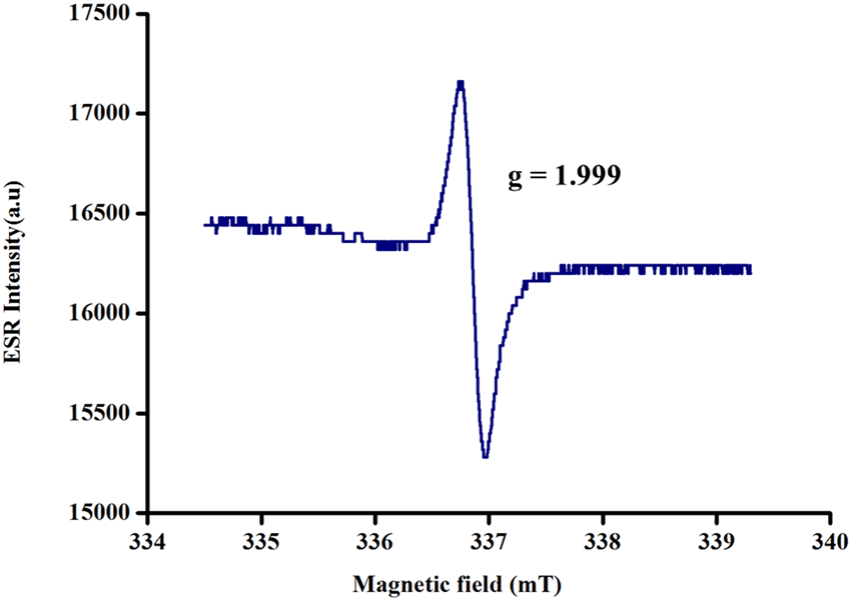
ESR spectrum of rGO–nAg recorded at room temperature.

The ability of rGO–nAg to form free radicles was demonstrated using ESR spectroscopy. As can be seen form the spectrum (Fig. 4), a strong single signal with a proportionality factor *g* value of approximately 2 (g = 1.99916) indicates the formation of oxygen-centred free radical^36, 37^. As the free radicles are very short lived, their direct visualization is difficult and requires the use of appropriate traps.

### Zone of inhibition

**Figure 5:**
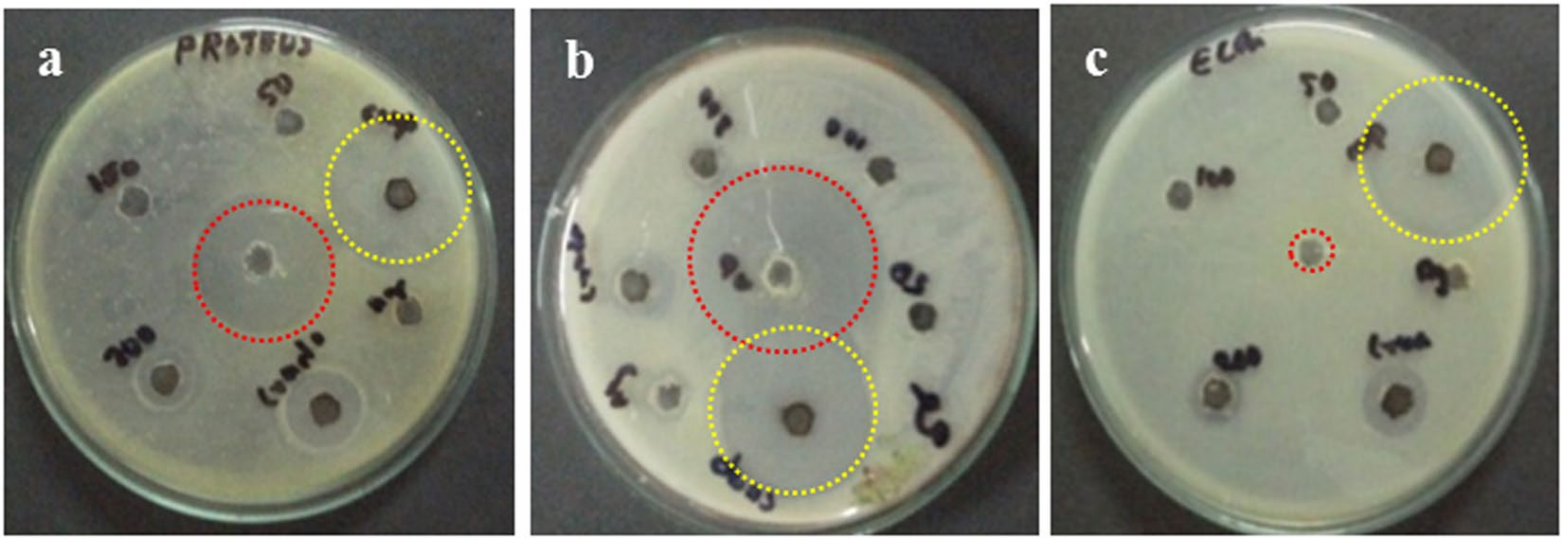
Well diffusion study. Representative plates of (**a**) *P. mirabilis*, (**b**) *S. aureus*, and (**c**) *E. coli*. Red circles indicate the zone of inhibition from wells loaded with nitrofurantoin; yellow circles indicate the zone of inhibition from wells loaded with rGO–nAg.

Plates were inoculated with *S*. *aureus*, *E*. *coli* and *P*. *mirabilis* and allowed to grow to achieve confluency. Wells containing various concentrations of rGO, nAg, rGO–nAg, or standard antibiotic nitrofurantoin were made in the plates. Zones of inhibition were measured after 24 hr. of incubation. Figure 5 shows representative images of plates.

### Survival rate vs time

**Figure 6:**
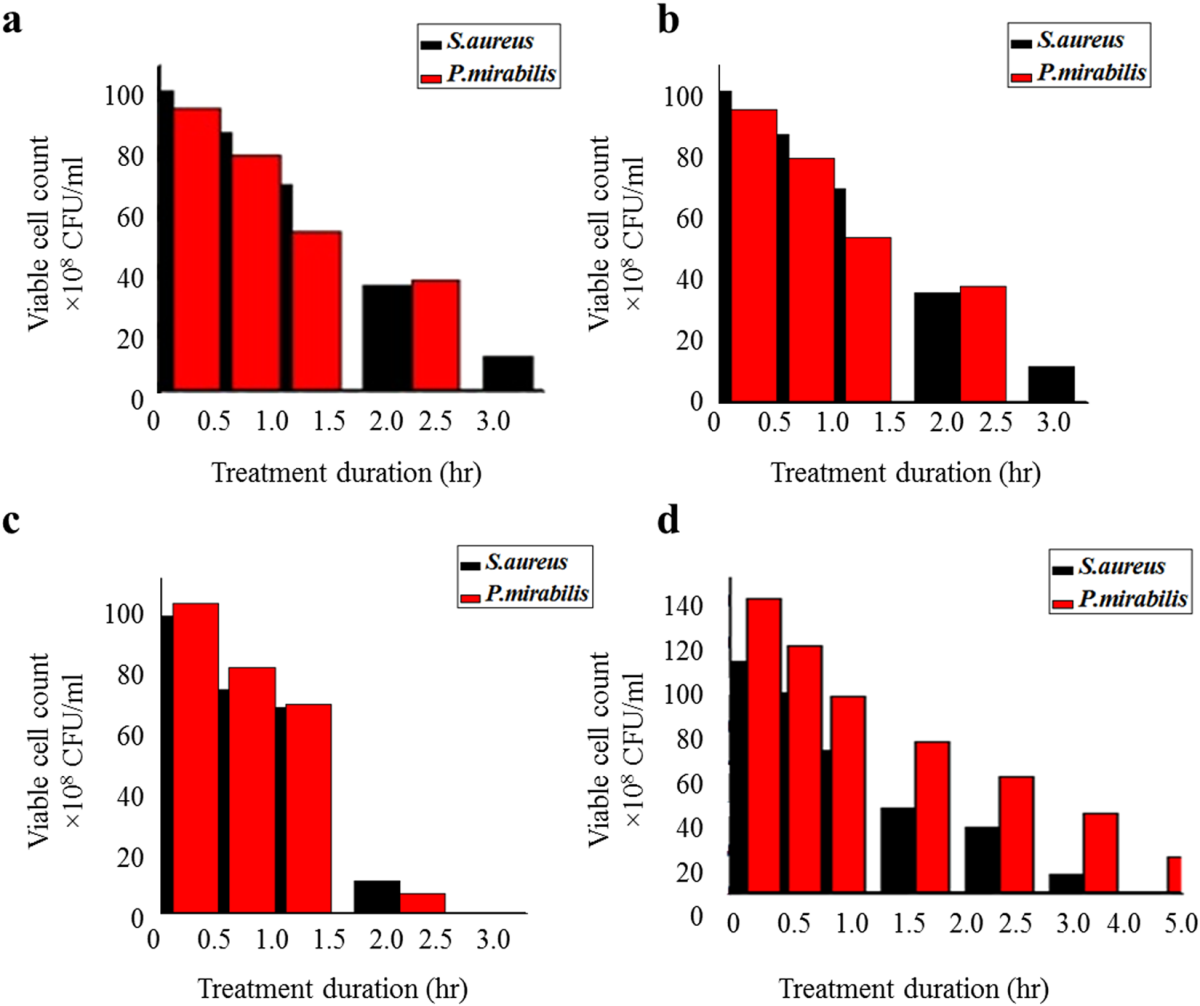
Viable count of bacteria after exposure to (**a**) rGO, (**b**) nAg, (c) rGO–nAg composite, and (**d**) standard antibiotic nitrofurantoin.

The survival rate vs time was calculated for both gram positive *S. aureus* and gram negative *P. mirabilis*. At the end of each exposure time of 24 hours, the samples were inoculated on plate count agar (PCA) and the results were tabulated as colony forming units (CFU/ml). Complete inhibition was detected at the end of 4 hours incubation in the presence of rGO and nAg, while rGO–nAg demonstrated complete inhibition at the end of 2–2.5 hours (Fig. 6).

## Discussion

Infections caused by multidrug resistant (MDR) isolates are usually difficult to treat. The pharmaceutical industry is now facing a great challenge due to the evolution of multidrug resistant and pandrug resistant organisms. The discovery of new effective antibacterial agents is challenging, time consuming (it could take well over 10 years from discovery to obtaining all regulatory approvals) and expensive. Nanoparticles may address this need and provide a novel therapeutic solution to limit the problem of antibiotic resistance^38^.

The antibacterial activity of rGO, nAg, and rGO–nAg composite was assessed against three important pathogenic bacterial species, namely *S*. *aureus, E. coli* and *P. mirabilis*. rGO exhibited considerable broad spectrum antibacterial activity against both Gram-positive *S*. *aureus* and Gram-negative *E. coli* and *P. mirabilis* bacterial pathogens, however it required a significantly higher concentration to achieve the desired level of inhibition compared to rGO–nAg. In the agar well diffusion method, rGO exhibited only a small zone of inhibition while rGO–nAg composite was able to achieve a zone of inhibition twice the size of rGO used on its own. It is important to note that inhibitory activity of rGO–nAg and rGO was observed against the multidrug resistant strain (resistant to more than three antibiotics, including nitrofurantoin) of *E. coli* used in this study. Time-resolved measurement of survival showed inhibition of *P. mirabilis* by rGO between 2–3 hr after exposure and complete inhibition after 3 hr of incubation. Similar results were shown for *S*. *aureus*, where significant inhibition was observed after 3 hr. After 18 hr of incubation, no viable organisms could be detected. The results obtained from this study correlated well with the previously published findings^39^.

Time-resolved viability testing showed that nAg significantly inhibited *P*. *mirabilis* after 3 hr and *E. coli* after 4 hr of exposure. Even though nAg could not inhibit the growth of either *P*. *mirabilis* or *E. coli* when tested using well method, the coupling of nAg with rGO significantly enhanced the inhibitory activity of the composite.

In addition to larger zone of inhibition, rGO–nAg composites significantly reduced time necessary to achieve complete inhibition. Both *S*. *aureus* and *P*. *mirabilis* were completely inhibited after 2.5 hr of incubation, with a significant reduction in the number of viable bacteria attained after 2 hr of incubation. The required incubation time for rGO–nAg was not only shorter than that required for either rGO or nAg, but also shorter than that required to achieve the same reduction in viability using standard antibiotic nitrofurantoin (100 µg/ml).

While nitrofurantoin kills the bacterial by damaging bacterial DNA^40^, the mechanism of activity of rGO–nAg is yet to be fully elucidated. Previous studies have suggested that one potential mode of action of sheet-like graphene-based materials involves cell physical wrapping and entrapment of bacterial cells by these nanomaterials. In addition to physical entrapment of the cell, the direct contact between the sharp edges of rGO sheets with cells can physically damage cell membrane, resulting in leakage of intracellular material and negatively affecting cell metabolism. The edge of graphene nanosheets have relatively high aspect ratio which makes them an attractive nanostructure for direct contact inactivation of microorganisms^20^. From this standpoint, increasing the sheet area enhances the rate of inactivation^41^.

Essential to cell growth and metabolism, bacterial respiration relies on electron transport mediated by extracellular electron acceptors^42^. An electron conduit that forms between surface respiratory proteins of the microbial membranes and the extracellular environment generates energy needed to support cell activity. When surface respiratory proteins that display n-type semiconducting behavior and a bandgap of ~2.6–3.1 eV^43^ come into contact with semi metallic materials such as rGO, where the oxygen percentage content is low, Shottky barrier is formed and electrons are transferred from cell membranes to rGO electron acceptors driven by Fermi level alignment^44^. Since bacteria strive to maintain a negative resting membrane potential by means of proton gradient, contact with rGO may lead to steady loss of electrons over time^44^. The value of the surface charge differs depending on the bacterial species, with Gram-negative *E. coli* having a less negatively charged surface compared to Gram-positive *S. aureus*, due to the former having the membrane isoelectric point pI = 4~5 and the latter having the pI of 2~3 under culture medium conditions^44^. The differences in surface electron states may account, at least in part, for the differences in inhibitory activity of rGO and rGO–nAg composites against Gram-positive and Gram-negative bacterial strains.

Oxidative stress induced by rGO nanosheets and nAg also play an important role^39^. rGO–nAg causes the oxidative stress by an imbalance between the production of reactive oxygen and the ability of the biological system (such as bacterial cell) to readily detoxify the reactive intermediates or easily repair the resulting damage^45^. The excess formation or insufficient removal of highly reactive molecules, such as reactive oxygen species (ROS), and resultant oxidative stress can arise from an increase in oxidant generation, a decrease in antioxidant protection, or a failure to repair oxidative damage^46^. This eventually leads to significant cell damage and cell death^47^.

Depending on their size and oxidation level, rGO sheets can adsorb on the surface layer of the cell, embed and subsequently cross the lipid bilayer, or can be taken up by the cell via vesicular structures^48^. Graphene sheets with higher degree of oxidation can enter the cell more efficiently owing to the lower energy state that exists between an oxidized graphene sheet and the membrane^49, 50^. It has been observed that the nature of the graphene edges, e.g. their sharpness and chemical composition, mediated the penetration of graphene in the lipid bilayers. The initial piercing of the cell membrane by sharp and rough edges of graphene has been shown to lower the energy barrier for graphene penetration^51^.

Treatment with nAg also contributes to oxidative stress through the formation of free radicals^52, 53^. Among generated reactive oxygen species (ROS), superoxide, hydrogen peroxide and hydroxyl radicals were reported to play key roles in the observed oxidative activity^54, 55^. The free radicals, which are short-lived reactive chemical intermediates that contain one or more unpaired electrons^56^, induce cellular damage when they pass this unpaired electron onto nearby cellular structures. This leads to oxidation of cell membrane lipids and amino acids that make up proteins or nucleic acid^57^. Ag ion treatment has been shown to result in cytoplasm membrane shinkage and separated from the cell wall. This led to release of cellular contents and significant cell wall degradation^58^. Similarly, reduced graphene oxide induces ROS-dependent oxidative stress by excess accumulation of intracellular ROS, such as hydrogen peroxide, superoxide anions, hydroxyl radicals and singlet molecular oxygen^49, 59, 60^. The ability of carbon nanostructures to generate oxygen anions and hydroxyl radicals were studied and confirmed by many researchers by employing ESR techniques^61–63^.

**Figure 7:**
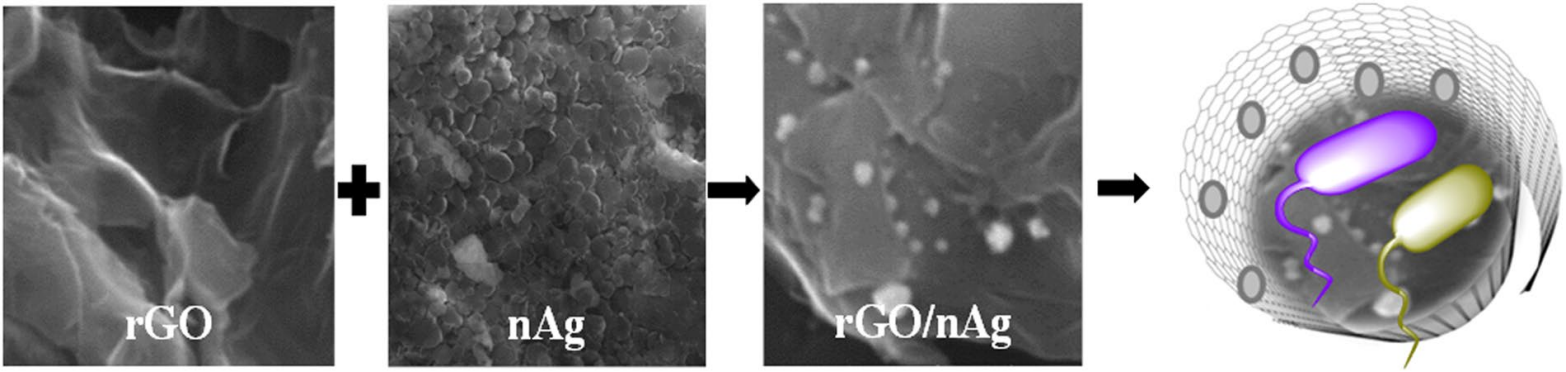
A symbolic representation of the mechanism of process of destruction of bacteria from the cumulative effect of cell-wrapping as well as cell - trapping mechanisms of rGO nanosheets and cell penetration of Ag nanomaterial.

The synergic effect of the individual components, nAg and rGO, as shown in Table 1 is responsible for the observed increase in antibacterial activity of rGO–nAg nanocomposite (Fig. 7)^64^. With regard to the rGO–nAg composite, physical interaction between the sharp edges of rGO sheets disrupts the cell membrane and facilitates the transport of silver ions across the cell membrane^65–68^. The cell entrapment property of rGO ensures high local concentrations of Ag ions in the immediate proximity of the cell membrane. It is also possible that rGO contributed to increased permeation of silver ions into the bacteria. Similar effects have been observed in Ag nanoparticles encapsulated in poly lactic acid polymer matrix, where lactic acid disrupted the bacteria cell membrane and thus facilitated entry of silver ions/nanoparticles into the Gram-positive and Gram-negative bacteria^69^.

**Table 1:** The average zones of inhibition (in mm) of rGO, nAg, rGO–nAg, and nitrofurantoin.

Isolates	*P. mirabilis*	*E. coli*	*S. aureus*
*rGO*			
50 µg/ml	No zone	No zone	No zone
100 µg/ml	No zone	No zone	No zone
200 µg/ml	18 ± 2	9 ± 1	No zone
*nAg*			
100 µg/ml	No zone	No zone	8 ± 1
*rGO*–*nAg*			
100 µg/ml	23 ± 2	25 ± 2	24 ± 1
*Nitrofurantoin*			
100 µg/ml	24 ± 2	No zone	26 ± 1

As expected, the zone of inhibition for rGO and nAg was concentration-dependent. The concentrations of 50 µg/ml and 100 µg/ml of rGO and nAg, respectively, were insufficient to inhibit the organisms tested. At these concentrations, nitrofurantoin inhibited *P. mirabilis* and *S. aureus*, but not *E. coli*. rGO–nAg nanomaterial composite showed strong activity, inhibiting all pathogens tested, including *E. coli* shown to be resistant to standard antibiotic.

An important characteristic of metals like silver is their capacity to participate in redox reactions. In addition to the affinity of a metal for a donor ligand, reduction potential is a thermodynamic parameter that determines the tendency of a metal species to acquire electrons from a donor and become reduced. The donor species loses electrons and becomes oxidized; thus, reduction and oxidation always occur simultaneously^70^.

Another destruction mechanism of rGO nano sheets is by extracting phospholipids from lipid membranes^51^. Graphene’s unique two-dimensional structure with all sp^2^ carbons facilitates strong dispersion interactions between graphene and cell membrane lipid molecules. On the surface of graphene cooperative movements of extracted lipid molecules were observed due to the redistribution of the hydrophobic tails to maximize hydrophobic interactions with the graphene surface. This lipid extraction mediated destructive method was demonstrated by previous research for both outer and inner membranes of *E. coli* ^51^. While exposure to rGO results in the dose-dependent loss of membrane integrity, as characterised by progressive extraction of adenine and protein from bacteria^71^, soft acids such as Ag tend to associate tightly with soft bases, such as the sulphhydryl (R–SH) groups that are found in proteins. Consequently, the antibacterial toxicity of these metals which is approximately proportional to their affinity for –S destructs the cells by protein denaturation^72, 73^. Moreover, pore formation can occur when all phospholipids are oxidized and this allows reactive oxygen species to enter the cell and cause oxidative damage to intracellular macromolecules, such as DNA or proteins. Previous research have also found that high concentration of reactive oxygen and nitrogen species are produced during the treatment of the cell membrane with plasma, an ionised gas consisting of highly reactive ions, electrons, photons and neutral species, and this can even destroy the cell membrane of cancer cells^74^.

The rGO–nAg composite may also disrupt the cellular donor ligands that coordinate Fe. The direct or indirect destruction of [4Fe–4S] clusters could result in the release of additional Fenton-active Fe into the cytoplasm, resulting in an increased ROS formation^70^. While at low doses, cells may be able to upregulate ROS-detoxification enzymes to withstand toxic doses of these elements, higher doses may inflict irreversible damage on cells.

**Figure 8:**
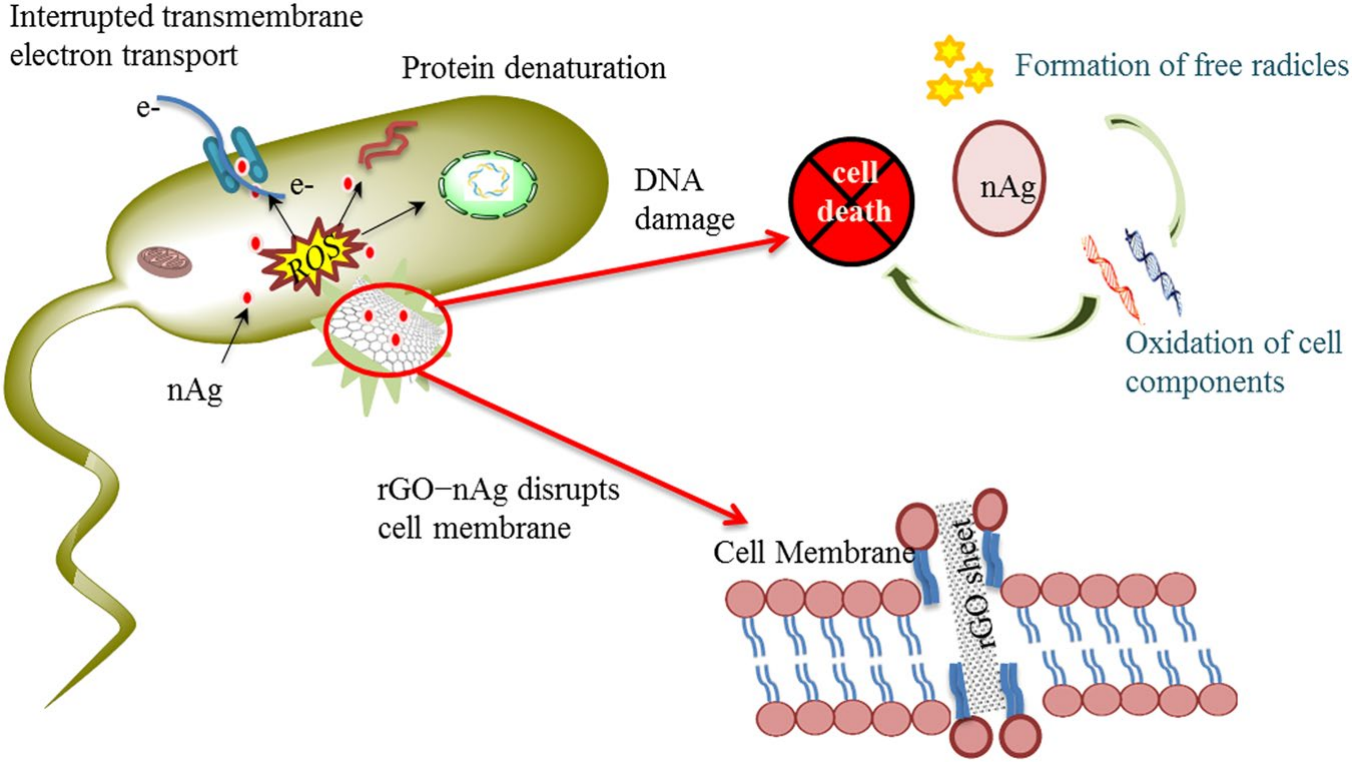
A symbolic representation of the mechanism by which the rGO–nAg nanoparticles kill the bacteria. The rGO punctures cell wall and enter the cytoplasm. Silver nanoparticles directly enter into the cell, induces oxidative stress and damage the cell contents.

Together, the cell membrane penetrating properties of rGO sheets, the oxidative stress of rGO and nAg and the free radicle formation of Ag nanoparticles contribute to enhanced antibacterial efficacy of rGO–nAg nanocomposites (Fig. 8).

The morphology of the cells plays a vital role in the bactericidal effect of rGO–nAg nanocomposite. Gram-positive and Gram-negative bacteria possess dissimilar cell wall structure and chemical composition^75^. In comparison with the delicate thin peptidoglycan cell membrane in Gram-negative bacteria, Gram-positive bacteria possess cell wall consisting of multiple layers of peptidoglycan which provide better cell membrane integrity and prevent cell disruption^76, 77^. Previously, exposure of *S. aureus* cells to rGO–nAg nanocomposite has been shown to result in cell wrinkling and damage, with some cells being completely covered by the rGO–nAg, whereas exposure of *E. coli* to the same concentrations of rGO–nAg led to complete cell fragmentation^77^. In other words, for Gram-negative *E. coli*, the primary mechanism of rGO–nAg bactericidal activity is through disruption of bacterial cell wall integrity, whereas for Gram-positive *S. aureus*, the effect is bacteriostatic and is associated with dramatic hindering of cell growth^77^.

## Conclusion

In this study, rGO and nAg nanomaterials were first synthesized using wet chemical methods, and then combined to form rGO–nAg nanocomposites. The properties of individual materials and the uniform distribution of nAg on rGO sheets were confirmed using microscopy and spectroscopy techniques. The produced rGO–nAg nanomaterial composites exhibited enhanced efficacy against all three pathogens tested. The activity of rGO–nAg nanocomposite was also superior to that of conventional systemic antibiotic, nitrofurantoin, even for a multidrug resistant strain of *E. coli* used in this study. The antibacterial activity of rGO–nAg composite against *S. aureus* is even more significant, being far superior to that of nitrofurantoin. These results suggest that rGO–nAg nanocomposite may present a viable alternative to some conventional antibacterial agents.

## Methods

### Synthesis and characterization of rGO, nAg, and rGO**–**nAg nanocomposite

Graphene oxide was prepared from natural graphite following Hummers method^78^. Briefly, 1 g of NaNO_3_ and 46 ml of H_2_SO_4_ was added to 1 g of natural graphite powder and stirred continuously in an ice bath to maintain the temperature of the mixture below 20 °C. Then, 6 g of KMnO_4_ was added slowly while stirring. After 1 hr, the ice bath was removed, the system was heated to 35 °C and the temperature was maintained at 35 °C for 30 min; 70 ml water was slowly added to the system and stirred for another 15 min. Then, 80 ml of hot (60 °C) water along with 30% H_2_O_2_ aqueous solution were added to reduce the residual KMnO_4_ until the bubbling has disappeared. The product formed was washed several times to remove the remaining salt impurities. After thermal reduction at 200 °C for 3 hr, a black colored powder of rGO was obtained.

AgNO_3_ was reduced by sodium potassium tartrate in the presence of poly vinyl pyrolidone (PVP) (MW 40,000) by first heating 50 ml solution of 1.2 mM PVP and 0.2 mM AgNO_3_ to 80 °C with vigorous stirring and then gradually adding 2 mM sodium potassium tartrate solution until complete reduction of AgNO_3_ had been achieved. Then the reaction mixture gradually became turbid and a light yellow suspension was obtained indicating the reaction was successful.

The rGO and nAg nanomaterial solutions were combined at the ratio of 9:1 using vigorous stirring for 2 hr, yielding rGO–nAg nanocomposite. By weight, nAg contributed 24% to the composite, estimated using EDS data.

The characterization of the synthesized composite was carried out using UV-Vis absorption spectroscopy, Fourier Transform Infrared (FTIR) spectrometry, X-Ray Diffraction Spectrometry (XRD), Scanning Electron Microscopy (SEM), Transmission Electron Microscopy (TEM) and Electron Spin Resonance (ESR) spectroscopy.

### Bacterial growth

The antibacterial activity of rGO, nAg and rGO–nAg nanocomposite was determined by modified agar well diffusion method^79^ and survival rate determination methods^80^. Clinical bacterial isolates of *S. aureus*, *P. mirabilis* and *E. coli* used in this study were obtained from Department of Microbiology, Vels University, Chennai, India, where they were extensively tested using standard methods for antibiotic susceptibility, e.g. using the double disc diffusion test and morphological characterization. Using Kirby-Bauer method, *E. coli* cultures isolated from UTI patients of a tertiary care hospital in Chennai were shown to be resistant to β-lactam antibiotics, such as ampicillin (10 μg/ml), attributed to the production of extended spectrum β-lactamases, and non-β-lactam antibiotics, such as gentamycin (10 μg/ml), co-trimoxazole (1.25/23.75 μg/ml), and ciprofloxacin (5 μg/ml)^81^. *S. aureus* isolates were found to be resistant to gentamycin (10 μg/ml), tetracycline (30 μg/ml), and trimoxozole (25 μg/ml), while being susceptible to chloramphenicol and ofloxacin at 30 and 32 μg/ml, respectively^82^. *P. mirabilis* was resistant to chloramphenicol (30 μg/ml), amoiclav (30 μg/ml), methicillin (30 μg/ml), and streptomycin (30 μg/ml), with susceptibility to ceffriaxone (30 μg/ml) and nalidixic acid (30 μg/ml)^83^.

The inocula for antibiogram assay were prepared following the recommendations of CLSI (2010 guidelines). Test organisms were incubated in standard nutrient broth at 37 °C for 4–6 hr. The inoculum, visual turbidity of 0.5 McFarland standards, was used to inoculate the surface of Mueller-Hinton agar plates. Wells of approximately 6 mm in diameter were made in the plates using a sterile borer. Each well was loaded with one of the following: undiluted rGO (crude), rGO solution (at 50 µg/ml, 100 µg/ml, or 200 µg/ml), nAg nanomaterial, or rGO–nAg nanocomposite. A standard antibiotic nitrofurantoin, an antibiotic clinically used for the treatment of these pathogens, (100 µg) was loaded in the center of the well to compare the antibacterial activity of the graphene composite. The plates were incubated at 37 °C for 18 hr.

The survival rates of gram-negative and gram-positive pathogens were determined using spread plate method. Three sets of flasks containing 100 ml of nutrient broth were inoculated with either *S. aureus* or *P. mirabilis* to the density of 3 × 10^8^ CFU/ml. To each flask, an antibacterial material, namely 0.1 g of rGO, 0.1 g of nAg, or 0.1 g of rGO–nAg was added. At regular time intervals, few ml aliquots of bacterial suspension were taken from each flask, and transferred onto agar plates, spread evenly and allowed to incubate for 18–24 hr at 37 °C, 5% CO_2_. The formed colonies were then counted using a plate counter.

## References

[1] News Release-“WHO’s first global report on antibiotic resistance reveals serious, worldwide threat to public health-30” *WHO Report* http://www.who.int/mediacentre/news/releases/2014/amr-report/en/ (2014).

[2] Bush K (2011). Tackling antibiotic resistance. Nature Rev. Microbiol..

[3] Levchenko I (2015). Plasma treatment for next-generation nanobiointerfaces. Biointerphases.

[4] Bazaka K, Jacob MV, Chrzanowski W, Ostrikov K (2015). Anti-bacterial surfaces: Natural agents, mechanisms of action, and plasma surface modification. RSC Adv..

[5] Hasan J, Chatterjee K (2015). Recent advances in engineering topography mediated antibacterial surfaces. Nanoscale.

[6] Leung E, Weil DE, Raviglione M, Nakatani H, On behalf of the World Health Organization World Health Day Antimicrobial Resistance Technical Working, G (2011). The WHO policy package to combat antimicrobial resistance. Bull. World Health Organ..

[7] Hegab HM (2016). The controversial antibacterial activity of graphene-based materials. Carbon.

[8] Hu W (2010). Graphene-based antibacterial paper. ACS Nano.

[9] Mejías Carpio IE, Santos CM, Wei X, Rodrigues DF (2012). Toxicity of a polymer-graphene oxide composite against bacterial planktonic cells, biofilms, and mammalian cells. Nanoscale.

[10] Nguyen Bich, H. & Nguyen Van, H. Promising applications of graphene and graphene-based nanostructures. *Adv. Nat. Sci-Nanosci*. **7** (2016).

[11] Sundramoorthy AK, Gunasekaran S (2014). Applications of graphene in quality assurance and safety of food. Trends in Anal. Chem..

[12] Bazaka K, Jacob MV, Ostrikov K (2016). Sustainable Life Cycles of Natural-Precursor-Derived Nanocarbons. Chem. Rev..

[13] Jacob MV (2015). Catalyst-Free Plasma Enhanced Growth of Graphene from Sustainable Sources. Nano Lett..

[14] Park S, Ruoff RS (2009). Chemical methods for the production of graphenes. Nat. Nano..

[15] Geim AK, Novoselov KS (2007). The rise of graphene. Nat. Mater..

[16] Pal S, Tak YK, Song JM (2007). Does the Antibacterial Activity of Silver Nanoparticles Depend on the Shape of the Nanoparticle? A Study of the Gram-Negative Bacterium *Escherichia coli*. Appl. Environ. Microbio..

[17] Pei S, Cheng H-M (2012). The reduction of graphene oxide. Carbon.

[18] Ivey KN (2008). MicroRNA Regulation of Cell Lineages in Mouse and Human Embryonic Stem Cells. Cell Stem Cell.

[19] Lerf A, He H, Forster M, Klinowski J (1998). Structure of Graphite Oxide Revisited. J. Phys. Chem. B.

[20] Akhavan O, Ghaderi E (2010). Toxicity of Graphene and Graphene Oxide Nanowalls Against Bacteria. ACS Nano.

[21] Eduok, S. *et al*. Insights into the effect of mixed engineered nanoparticles on activated sludge performance. *FEMS Microbiol. Ecol*. **91** (2015). 10.1093/femsec/fiv082 PMC4629872 26187478

[22] Schacht VJ (2013). Effects of silver nanoparticles on microbial growth dynamics. J. Appl. Microbiol..

[23] Beyth N, Houri-Haddad Y, Domb A, Khan W, Hazan R (2015). Alternative Antimicrobial Approach: Nano-Antimicrobial Materials. J. Evid. Based Complemen. Altern. Med..

[24] Clement JL, Jarrett PS (1994). Antibacterial Silver. Metal-Based Drugs.

[25] Kim JS (2007). Antimicrobial effects of silver nanoparticles. Nanomed. Nanotech. Biol. Med..

[26] Guzman M, Dille J, Godet S (2012). Synthesis and antibacterial activity of silver nanoparticles against gram-positive and gram-negative bacteria. Nanomed. Nanotech. Biol. Med..

[27] Liu J, Sonshine DA, Shervani S, Hurt RH (2010). Controlled Release of Biologically Active Silver from Nanosilver Surfaces. ACS Nano.

[28] Jain J (2009). Silver Nanoparticles in Therapeutics: Development of an Antimicrobial Gel Formulation for Topical Use. Mol. Pharm..

[29] Vasilev K (2010). Tunable Antibacterial Coatings That Support Mammalian Cell Growth. Nano Lett..

[30] Taheri S (2014). Substrate independent silver nanoparticle based antibacterial coatings. Biomaterials.

[31] Loryuenyong V, Totepvimarn K, Eimburanapravat P, Boonchompoo W, Buasri A (2013). Preparation and Characterization of Reduced Graphene Oxide Sheets via Water-Based Exfoliation and Reduction Methods. Adv. Mater. Sci. Eng..

[32] Abdolhosseinzadeh S, Asgharzadeh H, Seop Kim H (2015). Fast and fully-scalable synthesis of reduced graphene oxide. Sci. Rep..

[33] Xu C (2015). Fabrication and Characteristics of Reduced Graphene Oxide Produced with Different Green Reductants. PLoS One.

[34] Zainy M (2012). Simple and scalable preparation of reduced graphene oxide–silver nanocomposites via rapid thermal treatment. Mater. Lett..

[35] Marimuthu S (2012). Lousicidal activity of synthesized silver nanoparticles using Lawsonia inermis leaf aqueous extract against Pediculus humanus capitis and Bovicola ovis. Parasitol. Res..

[36] Tang H-R, Zhao B-L, Belton PS, Sutcliffe LH, Ng A (2000). Anomalous proton NMR relaxation behavior of cell wall materials from Chinese water chestnuts. Magn. Reson. Chem..

[37] Xiong L-B, Li J-L, Yang B, Yu Y (2012). In the Surface of Titanium Dioxide: Generation, Properties and Photocatalytic Application. J. Nanomater..

[38] Davies J (2007). Microbes have the last word. A drastic re-evaluation of antimicrobial treatment is needed to overcome the threat of antibiotic-resistant bacteria. EMBO Rep..

[39] Liu S (2011). Antibacterial Activity of Graphite, Graphite Oxide, Graphene Oxide, and Reduced Graphene Oxide: Membrane and Oxidative Stress. ACS Nano.

[40] Macrodantin (nitrofurantoi) *Hearst Magazine, UK* http://www.netdoctor.co.uk/medicines/infections/a7052/macrodantin-nitrofurantoin/ (2013).

[41] Perreault F, de Faria AF, Nejati S, Elimelech M (2015). Antimicrobial Properties of Graphene Oxide Nanosheets: Why Size Matters. ACS Nano.

[42] Lampa-Pastirk S (2016). Thermally activated charge transport in microbial protein nanowires. Sci. Rep..

[43] Eley DD, Spivey DI (1960). The semiconductivity of organic substances. Part 6-A range of proteins. T. Faraday Soc..

[44] Li J (2014). Antibacterial activity of large-area monolayer graphene film manipulated by charge transfer. Sci. Rep..

[45] Bansal, A. K. & Bilaspuri, G. S. Impacts of Oxidative Stress and Antioxidants on Semen Functions. *Vet. Med. Int*. **2011** (2011). 10.4061/2011/686137 PMC2943128 20871827

[46] Mohanty JG, Nagababu E, Rifkind JM (2014). Red blood cell oxidative stress impairs oxygen delivery and induces red blood cell aging. Front. Psychol..

[47] Rahman K (2007). Studies on free radicals, antioxidants, and co-factors. Clin. Interv. Aging.

[48] Perreault F, Fonseca de Faria A, Elimelech M (2015). Environmental applications of graphene-based nanomaterials. Chem. Soc. Rev..

[49] Zou X, Zhang L, Wang Z, Luo Y (2016). Mechanisms of the Antimicrobial Activities of Graphene Materials. J. Am. Chem. Soc..

[50] Li Y (2013). Graphene microsheets enter cells through spontaneous membrane penetration at edge asperities and corner sites. Proc. Natl. Acad. Sci. USA.

[51] Tu Y (2013). Destructive extraction of phospholipids from *Escherichia coli* membranes by graphene nanosheets. Nat. Nano.

[52] Fu PP, Xia Q, Hwang H-M, Ray PC, Yu H (2014). Mechanisms of nanotoxicity: Generation of reactive oxygen species. J. Food Drug Anal..

[53] Bhattacharyya A, Chattopadhyay R, Mitra S, Crowe SE (2014). Oxidative Stress: An Essential Factor in the Pathogenesis of Gastrointestinal Mucosal Diseases. Physiol. Rev..

[54] Zhang W, Li Y, Niu J, Chen Y (2013). Photogeneration of reactive oxygen species on uncoated silver, gold, nickel, and silicon nanoparticles and their antibacterial effects. Langmuir.

[55] Sondi I, Salopek-Sondi B (2004). Silver nanoparticles as antimicrobial agent: A case study on *E. coli* as a model for Gram-negative bacteria. J. Colloid Interface Sci..

[56] McDonnell G, Russell AD (1999). Antiseptics and Disinfectants: Activity, Action, and Resistance. Clin. Microbiol. Rev..

[57] Maliszewska I, Sadowski Z (2009). Synthesis and antibacterial activity of of silver nanoparticles. J. Phys. Conf. Ser..

[58] Jung WK (2008). Antibacterial Activity and Mechanism of Action of the Silver Ion in Staphylococcus aureus and *Escherichia coli*. Appl. Environ. Microbiol..

[59] Lukowiak A, Kedziora A, Strek W (2016). Antimicrobial graphene family materials: Progress, advances, hopes and fears. Adv. Colloid Interface Sci..

[60] Szunerits S, Boukherroub R (2016). Antibacterial activity of graphene-based materials. J. Mater. Chem. B.

[61] Ge C (2012). The contributions of metal impurities and tube structure to the toxicity of carbon nanotube materials. NPG Asia Mater..

[62] Zhang W (2012). Graphene: Unraveling Stress-Induced Toxicity Properties of Graphene Oxide and the Underlying Mechanism. Adv. Mater..

[63] He W, Liu Y, Wamer WG, Yin J-J (2014). Electron spin resonance spectroscopy for the study of nanomaterial-mediated generation of reactive oxygen species. J. Food Drug Anal..

[64] Barua S (2014). One step preparation of a biocompatible, antimicrobial reduced graphene oxide–silver nanohybrid as a topical antimicrobial agent. RSC Adv..

[65] Tang X-Z, Chen X, Wu G, Hu X, Yang J (2015). Improved chemical stability of silver by selective distribution of silver particles on reduced graphene oxide nanosheets. RSC Adv..

[66] Tian T (2014). Graphene-Based Nanocomposite As an Effective, Multifunctional, and Recyclable Antibacterial Agent. ACS Appl. Mater. Interfaces.

[67] Ocsoy I (2013). Nanotechnology in plant disease management: DNA-directed silver nanoparticles on graphene oxide as an antibacterial against Xanthomonas perforans. ACS Nano.

[68] Das MR (2011). Synthesis of silver nanoparticles in an aqueous suspension of graphene oxide sheets and its antimicrobial activity. Colloids Surf., B.

[69] Shima T (2014). Synthesis and surface immobilization of antibacterial hybrid silver-poly(l-lactide) nanoparticles. Nanotechnology.

[70] Lemire JA, Harrison JJ, Turner RJ (2013). Antimicrobial activity of metals: mechanisms, molecular targets and applications. Nat. Rev. Microbiol..

[71] Nanda SS, Yi DK, Kim K (2016). Study of antibacterial mechanism of graphene oxide using Raman spectroscopy. Sci. Rep..

[72] Workentine ML, Harrison JJ, Stenroos PU, Ceri H, Turner RJ (2008). Pseudomonas fluorescens’ view of the periodic table. Environ. Microbiol..

[73] Nies DH (2003). Efflux-mediated heavy metal resistance in prokaryotes. FEMS Microbiol. Rev..

[74] Van der Paal J, Neyts EC, Verlackt CCW, Bogaerts A (2016). Effect of lipid peroxidation on membrane permeability of cancer and normal cells subjected to oxidative stress. Chem. Sci..

[75] Silhavy TJ, Kahne D, Walker S (2010). The Bacterial Cell Envelope. Cold Spring Harb. Perspect. Biol..

[76] Tang J (2013). Graphene Oxide–Silver Nanocomposite As a Highly Effective Antibacterial Agent with Species-Specific Mechanisms. ACS Appl. Mater. Interfaces.

[77] Geetha Bai R (2016). The biogenic synthesis of a reduced graphene oxide-silver (RGO-Ag) nanocomposite and its dual applications as an antibacterial agent and cancer biomarker sensor. RSC Adv..

[78] Cao N, Zhang Y (2015). Study of Reduced Graphene Oxide Preparation by Hummers; Method and Related Characterization. J. Nanomater..

[79] Balouiri M, Sadiki M, Ibnsouda SK (2016). Methods for *in vitro* evaluating antimicrobial activity: A review. J. Pharma. Anal..

[80] Girija SA, Priyadharshini VJ, Suba PK, Hariprasad P, Raguraman R (2012). Antibacterial effect of squid ink on ESBL producing strains of *Escherichia coli* and Klebsiella pneumoniae. Indian J. Geomarine Sci..

[81] Gururajan G, Kaliyaperumal KA, Ramasamy B (2011). Prevalence of extended spectrum beta lactamases in uropathogenic *Escherichia coli* and Klebsiella species in a Chennai suburban tertiary care hospital and its antibiogram pattern. J. Microbiol..

[82] Fayaz M, Sivakumaar PK, Joe MM (2014). Prevalence and Antibiotic Susceptibility Pattern of Dental Biofilm forming Bacteria. Int. J. Curr. Microbiol. App. Sci.

[83] Syntem S, Dutta H, Kalyani M (2016). Characterization of Proteus Species and Detection of Multi Drug Resistant (MDR) with Special Reference to ESBL Strains. Int. J. Curr. Microbiol. App. Sci..

